# Evolution of Discourse in the Reddit Hemorrhoid Community: Topic and Sentiment Analysis

**DOI:** 10.2196/90427

**Published:** 2026-09-21

**Authors:** Zhiguang Huang, Xinchang Liu, Zhenjie Yu, Yan He, Xinyao Yi, Hei Hang Edmund Yiu, Anyu Gao, Wai-kit Ming

**Affiliations:** 1 Department of Infectious Diseases and Public Health, Jockey Club College of Veterinary Medicine and Life Sciences, City University of Hong Kong, To Yuen Building, 31 To Yuen Street, Hong Kong, China (Hong Kong), 852 34426956; 2 Centre for Safe Medication Practice and Research, Department of Pharmacology and Pharmacy, LKS Faculty of Medicine, The University of Hong Kong, Hong Kong, China (Hong Kong); 3 Cambridge Judge Business School, University of Cambridge, Cambridge, United Kingdom; 4 Faculty of Business and Economics, The University of Hong Kong, Hong Kong, China (Hong Kong); 5 Institute of Global Governance and Innovation for a Shared Future, City University of Hong Kong, Hong Kong, China (Hong Kong)

**Keywords:** online health communities, hemorrhoidal disease, topic modeling, emotion analysis, social support, Reddit, stigmatized illness

## Abstract

**Background:**

Online health communities are crucial resources for individuals managing socially stigmatized and physically burdensome conditions, such as hemorrhoids. These anonymous forums provide critical avenues for emotional validation and information exchange that may be underrepresented or difficult to access in traditional clinical settings. Despite their growing importance as data sources for understanding patient experiences, large-scale longitudinal analyses of the thematic and emotional evolution within such communities remain scarce.

**Objective:**

This study aimed to conduct a longitudinal computational analysis of the r/hemorrhoids Reddit community (2013‐2022) to characterize its thematic concerns, examine its emotional landscape, and explore how emotional expression relates to community engagement.

**Methods:**

We collected 9954 Reddit posts, which were refined through preprocessing into a final dataset of 3746 high-quality posts. Our methodology involved a multistage computational approach: (1) topic modeling was performed on the mature phase of the community (2019‐2022) using BERTopic to identify stable thematic clusters; (2) fine-grained emotion classification used a DistilRoBERTa-based transformer model (j-hartmann/emotion-english-distilroberta-base) to categorize posts into 7 discrete emotional states (neutral, fear, sadness, joy, anger, surprise, and disgust); and (3) longitudinal analyses examined temporal trends in topic prevalence during the mature phase (2019‐2022) and emotional distributions across the full study period (2013‐2022), while also assessing associations between emotional categories and engagement metrics (post score and comment count).

**Results:**

The analysis revealed a polarized emotional distribution dominated by neutral (n=1247, 33.29%) and high levels of negative affect (fear and disgust). Thematic modeling identified four higher-order thematic domains encompassing 19 semantically coherent topics: (1) symptom recognition and diagnosis; (2) conservative treatment and self-management; (3) medical and surgical interventions; and (4) quality of life, risk factors, and psychosocial impact. Posts expressing fear and sadness received the highest mean comment counts (7.06, SD 7.60, and 7.03, SD 6.36, respectively), exceeding those expressing neutral or positive emotions.

**Conclusions:**

The findings suggest that the Reddit hemorrhoid community serves as an important platform for information exchange and emotional expression among individuals with a stigmatized condition. Posts expressing fear and sadness were associated with higher levels of community engagement, highlighting the potential value of online health communities in supporting patient communication and informing future digital health interventions.

## Introduction

Online health communities (OHCs) have emerged as critical ecosystems for individuals navigating complex health journeys, offering informational and emotional support that complements traditional physician-patient interactions [1,2]. OHCs also play a critical role in fostering peer-to-peer empathy and social connectedness, which are increasingly recognized as key determinants of sustained patient engagement [3,4]. These digital forums are particularly vital for patients with conditions associated with social stigma or embarrassment, such as hemorrhoids and other anorectal diseases. Such patients may be hesitant to seek timely medical advice or openly share their experiences in offline settings [5]. Within the anonymous environments of platforms such as Reddit, users generate vast quantities of unsolicited, longitudinal data that authentically capture the concerns, challenges, and lived experiences often left unspoken in clinical encounters [6,7].

Given the potential of such platforms to reveal authentic patient concerns, hemorrhoids represent a particularly compelling case for study. Although hemorrhoids are among the most prevalent anorectal conditions worldwide and carry significant psychological and quality of life burdens [8,9], there remains a notable research gap concerning large-scale, longitudinal analyses of public discourse surrounding this condition. While existing research has leveraged social media to explore various health topics [6,7], the specific thematic concerns and emotional landscape within the hemorrhoid community remain largely unexplored. Previous investigations into stigmatized illnesses have predominantly relied on surveys and interviews—methodologies that are not only resource-intensive but may also fail to capture the spontaneous and candid nature of online discourse [10]. Recent advances in natural language processing (NLP) have enabled nuanced detection of emotional cues and empathetic expressions in OHC discourse, offering new opportunities to systematically examine how patients articulate their distress and seek support in online spaces [11,12].

This study aimed to address this research gap by conducting a comprehensive computational analysis of longitudinal text data collected from the Reddit community r/hemorrhoids between 2013 and 2022. Specifically, we sought to (1) identify the principal themes and core concerns within the community; (2) characterize the emotional landscape associated with these discussions; and (3) examine the relationship between thematic concerns, emotional expression, and community engagement. The findings provide data-driven insights into the informational and emotional needs of individuals living with hemorrhoids and may help inform clinician-patient communication, digital health interventions, and public health education.

## Methods

### Data Collection

This study used a large-scale longitudinal dataset derived from the publicly available Pushshift Reddit archive. Pushshift provides historical Reddit data collected via the Pushshift API and includes all publicly posted content from the r/hemorrhoids subreddit since its inception. Pushshift’s ability to receive new data was terminated around April 2023 following Reddit’s sweeping changes to its API and data access policies. Accordingly, we accessed records from this archive up until early 2023, subsequently filtering the content to a continuous time frame spanning November 10, 2013, to December 31, 2022. This process yielded our longitudinal research dataset. The initial data collection comprised 9954 posts. The primary analytical goal was to leverage this post text to gain comprehensive insights into the core issues and concerns initiated by users within the community.

### Data Preprocessing and Corpus Construction

To construct a high-quality corpus suitable for advanced NLP, we implemented a rigorous multistage data cleaning and filtering pipeline, following established preprocessing techniques in NLP research [13]. The initial collection of 9954 posts underwent systematic refinement. First, we excluded content identified as deleted or removed (ie, posts with the author tag “[deleted]” or the body text “[removed]”), which reduced the working dataset to 5836 posts. Next, we eliminated posts that lacked essential textual content, such as those with empty or whitespace-only selftext fields, resulting in 3963 posts. To ensure adequate linguistic richness for computational analysis, we applied a content-length threshold and removed posts containing fewer than 10 words in the selftext, consistent with previous social media topic modeling studies that excluded very short texts [14]. After applying this filtering criterion, 3872 posts remained.

Finally, we excluded posts exhibiting virtually no observable community response, defined as those with both a Reddit score of ≤1 and zero comments. This conservative conjunction (AND) criterion removed only posts with minimal evidence of community interaction while retaining low-score posts that nonetheless generated discussion or community endorsement. This step excluded 126 of 3872 posts (3.3% of the preprocessed corpus). After this 4-step refinement, we obtained a final dataset of 3746 high-quality posts. The analytical corpus was constructed by concatenating the title and body text of these posts, forming a cohesive document set that captures discourse spanning the entire study period. A detailed preprocessing flowchart is presented in Figure S1 in Multimedia Appendix 1.

### Topic Modeling

To identify the principal themes and concerns discussed within the community, we used the BERTopic modeling framework, an embedding-based topic modeling approach that has demonstrated improved thematic coherence compared with traditional methods such as latent Dirichlet allocation [15,16]. Prior to modeling, we conducted a preliminary longitudinal analysis of the dataset to examine the evolution of community activity. This analysis revealed a critical inflection point: post volume in the r/hemorrhoids subreddit exhibited significant exponential growth starting in 2019, marking a transition from a sparsely populated early formation stage to a highly active, mature stage of user interaction. This discovery directly informed our modeling strategy. As the early-stage data (2013‐2018) contained only 16 posts after preprocessing, the sample size was insufficient to support robust and credible quantitative topic modeling. Consequently, to ensure the depth and validity of the analysis, we strategically focused our topic modeling efforts on the community’s mature stage (2019‐2022), which encompassed 3730 high-quality posts and comprehensively reflected the discourse characteristics during the period of highest activity. At the same time, our longitudinal emotional trend analysis encompassed the entire study period (2013‐2022), thereby preserving the broader longitudinal perspective of the study. We implemented a rigorous BERTopic modeling pipeline for this mature-stage corpus. First, each document (the concatenated title and body text) was converted into a high-dimensional semantic vector using the “all-MiniLM-L6-v2” model from the SentenceTransformer library (Hugging Face). Subsequently, Uniform Manifold Approximation and Projection was applied to reduce the dimensionality of these embedding vectors to 5 (n_components=5), thereby preserving the crucial thematic structure. We then applied the Hierarchical Density-Based Spatial Clustering of Applications with Noise algorithm, setting the minimum cluster size to 20 (min_cluster_size=20) to identify document clusters of substantial scale, representing the emerging topics. To enhance topic quality and interpretability, we manually constructed a custom English stopword list based on the standard scikit-learn library (scikit-learn developer community). This list was iteratively refined and applied to remove general-purpose terms that were irrelevant to the study’s thematic focus. Following the extraction of preliminary keyword lists for each topic via the class-based term frequency–inverse document frequency (c-TF-IDF) algorithm, we further implemented a rigorous keyword standardization and refinement process to bolster thematic semantic consistency and interpretability. This process primarily involved merging synonyms and performing lexical normalization on the keywords. Specifically, following an iterative refinement process based on corpus inspection, we devised a custom mapping lexicon to semantically normalize the extracted keywords. This involved manual lemmatization and canonicalization, such as consolidating plurals to singulars (eg, “hemorrhoids” normalized to “hemorrhoid”), mapping distinct verbal and adjectival forms to their nominal or stem forms (eg, “removed,” “remove” standardized as “removal”; “itchy” standardized as “itch”), and unifying spelling variations and quasi-synonyms (eg, “haemorrhoid” normalized to “hemorrhoid”; “blood” standardized as “bleeding”). Upon application of these rules, the c-TF-IDF scores of the consolidated keywords were aggregated, forming a new, composite score. We then reranked the keywords for each topic based on this new score, selecting the top 5 as the most representative core terms. This critical step ensured that the final keyword lists used for manual interpretation were highly refined and semantically focused. All final generated topics were subjected to meticulous manual review by the research team based on their representative keywords and constituent documents. During this stage, Gemini 2.5 Pro (Google) was used solely to generate candidate descriptive labels based on the BERTopic-generated topic names and representative keywords. These model-generated labels served only as preliminary suggestions and were not automatically adopted. All candidate labels were independently reviewed and refined by the research team, and final topic labels were determined through discussion and consensus to ensure semantic accuracy and interpretive rigor. The topic representations reported in Table 1 reflect the original c-TF-IDF outputs prior to lexical standardization, whereas all downstream analyses and thematic interpretations were based on the refined and semantically normalized top-five keyword sets. To further evaluate topic quality, we calculated topic coherence (C_v) and topic diversity metrics based on the final BERTopic solution. Topic coherence assesses the semantic consistency of topic keywords, whereas topic diversity measures the distinctiveness of topic representations across the model.

**Table 1.** Summary and thematic organization of core user concerns identified by topic modeling (2019‐2022).

Topic	Count	Name^a^	Custom name^b^	Representation	Representative_Docs
0	444	0_cream_hemorrhoid_external_hemorrhoids	Topical treatments and ointments	“cream,” “hemorrhoid,” “external,” “hemorrhoids,” “pain,” “hydrocortisone,” “itching,” “internal,” “oil,” “doctor”	“I got hemorrhoid cream for my internal hemorrhoid that would prolapse out every time I pooped. ... after using the cream for 2 days, I took a poop today and checked... I didn’t see my inflamed, bulging red internal hemorrhoid at all. Does the cream remove my hemorrhoid or just shrink it?” “I’ve tried hydrocortisone cream, prep H cream, suppositories, and sitz baths. Doesn’t work... I have the same 2 external hemorrhoids. I’m trying the Chinese mayolong cream from today.”
1	359	1_blood_bleeding_stool_red	Bleeding symptoms and diagnosis	“blood,” “bleeding,” “stool,” “red,” “toilet,” “colonoscopy,” “hemorrhoids,” “bright,” “internal,” “paper”	“I would wipe my butt and see a quick little line of red on toilet paper.” “Never seen any blood coat or be mixed in the stool. The rest was on the toilet paper.” “I had a large amount of blood when wiping bright red, no pain, the most I’ve ever seen, pretty heavy.”
2	329	2_surgery_pain_hemorrhoidectomy_recovery	Hemorrhoidectomy: surgery and recovery	“surgery,” “pain,” “hemorrhoidectomy,” “recovery,” “op,” “bowel,” “bm,” “painful,” “better,” “hours”	“Operation was done in the early morning and was 100% painless. ... after I woke up, I had minor pain in my rectum, but nothing too bad. ... moderate 6/10 level pain kicked in after the anesthesia wore off.” “Get a routine of taking your pain meds on time. Do not worry about taking them a little early if need be. It is better to keep the pain away, rather than fight it.”
3	239	3_banding_band_rubber_procedure	Rubber band ligation procedure	“banding,” “band,” “rubber,” “procedure,” “ligation,” “banded,” “pain,” “internal,” “hemorrhoids,” “fall”	“I had 3 internal hemorrhoids identified by a colorectal surgeon, and he decided rubber band ligation would be the best treatment.” “The first one I had done was painful for about 48 hours. No real pain after that. went back 2 weeks later to have another one done, and that was about the same.” “So I had my second banding done yesterday, three bands. pain was about as bad, if not worse, than the first time.”
4	170	4_fiber_water_diet_eat	Dietary and lifestyle management	“fiber,” “water,” “diet,” “eat,” “hemorrhoids,” “poop,” “stool,” “lot,” “constipation,” “food”	“We talked about fiber and hydration and otc options. So I did all that, pretty religiously, for a while. I increased my fiber intake. started drinking way more water.” “I have had little fiber intake, combined or not enough water intake.”
5	168	5_anus_lump_hemorrhoid_blood	Physical symptoms: lumps and bumps	“anus,” “lump,” “hemorrhoid,” “blood,” “small,” “hard,” “hemorrhoids,” “pain,” “butt,” “noticed”	“I’ve had this soft flesh bump in my anus for a while. for months, actually, and it did get bigger over time.” “A small lump beside my anus (not on it).” “Imagine if a small part of your anus got swollen? and it’s making the outside part of the anus on top of it... protrude into a flappy bit that I could literally push inside, but it keeps coming out.”
6	126	6_thrombosed_hemorrhoid_pain_external	Thrombosed external hemorrhoids	“thrombosed,” “hemorrhoid,” “pain,” “external,” “clot,” “painful,” “size,” “doctor,” “bleeding,” “blood”	“lo and behold I have a huge external hemorrhoid... my entire left side.” “I notice that part of it, maybe 20% is purple/blue. oh boy, this is a thrombosed hemorrhoid I think.” “developed very large and painful thrombosed hemorrhoid.”
7	89	7_skin_tag_tags_removal	Anal skin tags and removal	“skin,” “tag,” “tags,” “removal,” “removed,” “swelling,” “anal,” “surgery,” “left,” “recovery”	“2 months later with ‘swelling’ still on my butt hole, I went back... and what do you know.... now my internal hemorrhoid is ‘pulling the skin around my anus’ or whatever that means - basically it’s prolapsed.” “hemorrhoidectomies are known to leave you a little present post procedure called... skin tags. I’m literally going in a f-word circle.”
8	86	8_hemorrhoids_internal_feeling_pressure	Internal hemorrhoids: sensation and pressure	“hemorrhoids,” “internal,” “feeling,” “pressure,” “pain,” “external,” “feels,” “hemorrhoid,” “cause,” “anus”	“The past week, I have a weird feeling like I have to go to the bathroom, but I can’t.” “It feels like something is in there.” “It’s like a feeling of pressure, fullness, or something.”
9	51	9_gym_lifting_exercises_exercise	Impact of exercise and weightlifting	“gym,” “lifting,” “exercises,” “exercise,” “weight,” “running,” “squats,” “weights,” “heavy,” “working”	“I’ve heard that lifting heavy weights at the gym and certain workouts like squats and heavy bench presses worsen the condition. how true is that?” “obviously there is a chance this caused my hem.” “the pressure on my lower abs caused this?”
10	50	10_hemorrhoids_water_fiber_diet	Comprehensive home remedies	“hemorrhoids,” “water,” “fiber,” “diet,” “sitz,” “eat,” “bowel,” “fasting,” “bath,” “eating”	“chipotle. big no no. All that sodium ramped up my hemorrhoids once again.” “I don’t think milk is a smart move.” “epsom salt baths - a wonderful pain remedy.” “I sat on ice packs and then switched to pillows (no ring pillows).” “I used cold water to clean back there... I use a bidet. ... hair dryer. You want to blow dry on cool mode after you’re done showering or using the sitz bath.”
11	47	11_bath_sitz_epsom_baths	Sitz baths and Epsom salts	“bath,” “sitz,” “epsom,” “baths,” “cold,” “water,” “salts,” “salt,” “hot,” “toilet”	“Is epsom salt the best to soak in a sitz bath twice a day with warm water?” “I’ve been taking sitz baths in my bath tub for my external hemorrhoid. however, cleaning the bath tub, filling it in with water each time was very time consuming.”
12	36	12_sitting_chair_sit_cushions	Sitting posture and support cushions	“sitting,” “chair,” “sit,” “cushions,” “donut,” “cushion,” “chairs,” “position,” “piles,” “best”	“Whenever I sit on a soft chair my internal hemorrhoids always seem to get worse.” “the softer the chair the more your ass sinks into the chair... and therefore more pressure is exerted on your hemorrhoids.” “I’ve discovered that sitting on a hard bench for a while... i get a not insignificant amount of relief.”
13	34	13_laser_surgery_hemorrhoids_treatment	Alternative surgical procedures	“laser,” “surgery,” “hemorrhoids,” “treatment,” “lis,” “surgeon,” “hospital,” “procedure,” “pain,” “india”	“laser is very much less painful than open surgery.” “the median postoperative pain score was 2.9 (range, 1‐5) with rubber band ligation vs 1.1 (range, 0‐2) for hemorrhoid laser procedure.” “resolution of symptoms was observed in 16 patients (53%) with ligation vs 27 (90%) with hemorrhoid laser procedure.”
14	31	14_masturbation_sex_hemorrhoids_congestion	Sexual activity and pelvic congestion	“masturbation,” “sex,” “hemorrhoids,” “congestion,” “venous,” “anal,” “cause,” “impaired,” “porn,” “return”	“My hemorrhoids definitely felt a little more inflamed during the masturbation week.” “a couple days with no fap and it felt back to normal.” “masturbated about 10 times in one day (a lot I know) and it started to physically hurt my butt when I orgasmed. my anus was sore for about a week and a half straight.”
15	29	15_fissure_diltiazem_pain_anal	Anal fissures and diltiazem treatment	“fissure,” “diltiazem,” “pain,” “anal,” “fissures,” “hemorrhoid,” “haemorrhoid,” “internal,” “healed,” “hemorrhoids”	“I also have a anal fissure... the pain as discomfort have been increasing slightly.” “I never experienced any bleeding but I did experience the other symptoms most have with hemorrhoids and fisusures.” “The diltiazem helps, but as one fissure heals, and I start to feel better, another one in another location shows up.”
16	26	16_thd_hal_surgery_rar	Minimally invasive surgery (THD^c^and HAL-RAR^d^)	“thd,” “hal,” “surgery,” “rar,” “procedure,” “halo,” “traditional,” “surgeon,” “journal,” “paid”	“The hal procedure is basically them shoving a device up you that detects the blood vessels and base of the location that causes the hemmies and suturing it shut.” “the thd surgery is basically the same thing as the halo surgery, but are both given different names.”
17	24	17_smell_odor_discharge_clean	Odor, discharge, and associated symptoms	“smell,” “odor,” “discharge,” “clean,” “smells,” “stink,” “mucus,” “scrotum,” “nifedipine,” “hemorrhoids”	“I keep on getting a really bad odor down there, but every time I take a shower and clean myself down there it still smells but I don’t personally smell it but other people do.” “Occasionally ill notice a spotting of what seems like residual stool but never more than a dime or sometimes hardened mucus that looks jelly like in tiny amounts.”
18	22	18_sex_anal_hemorrhoidectomy_hemorrhoid	Post-hemorrhoidectomy sexual activity	“sex,” “anal,” “hemorrhoidectomy,” “hemorrhoid,” “stenosis,” “vaginal,” “surgery,” “external,” “quite,” “bottoming”	“My main concern is how long it might take to heal enough in order to have vaginal sex somewhat comfortably, and also really enjoy it.” “I am having the crh banding done soon and am wondering... will I be able to have ’better’ anal sex after this?”

a Original topic labels generated automatically by BERTopic.

b Descriptive labels refined with the assistance of a large language model (Gemini 2.5 Pro) and finalized through expert review.

c THD: transanal hemorrhoidal dearterialization.

d HAL-RAR: hemorrhoidal artery ligation and recto-anal repair.

### Sentiment and Emotional Dynamics Analysis

To conduct a deeper, more granular emotional analysis of the community discourse, we performed emotion recognition on every post within the final full corpus (N=3746). This study moved beyond simple 3-class polarity classification comprising positive, negative, and neutral categories, using an advanced multiclass emotion recognition model. Specifically, we used the j-hartmann/emotion-english-distilroberta-base model (developed by Jochen Hartmann and hosted on the Hugging Face model repository), a DistilRoBERTa-based transformer model fine-tuned for the detection of subtle emotions [17]. This model can classify text into 7 independent emotion categories: joy, sadness, anger, fear, disgust, surprise, and neutral. When applying the model, we accounted for the input length constraint inherent to transformer architectures. For documents exceeding the maximum input length (512 tokens), we adopted the standard head truncation strategy, analyzing only the initial portion of the text for emotional content. This strategy is commonly adopted in large-scale emotion analysis of long-form social media posts and retains the most contextually salient emotional cues, which are often introduced early in user narratives [18,19]. This comprehensive procedure enabled us to achieve a dual analytical objective: first, to quantify the overall emotional landscape of the community across the entire study period (2013‐2022); and second, to deeply profile the emotional composition of each specific topic identified by the BERTopic model during the mature stage (2019‐2022), thereby uncovering the dynamic interplay between distinct concerns and their corresponding emotional expressions.

### Temporal Segmentation for Emotion Comparison

Given that the COVID-19 pandemic profoundly reshaped online health communication, we defined 2 analytically meaningful and duration-balanced time intervals to capture structural shifts in community activity. The pandemic surge period (January 2020 to January 2021) corresponds to the phase of rapid and sustained growth in posting volume at the onset of the global outbreak (n=1032 posts). The post-surge stabilization period (June 2021 to June 2022) reflects a later stage of elevated yet fluctuating engagement, during which pandemic-related online activity began to stabilize (n=1144 posts). These analytic windows were implemented using end-exclusive upper date boundaries.

The intervening months (February to May 2021) showed a comparatively flat and transitional pattern without a distinct structural trajectory; including this plateau phase would have diluted the contrast between the 2 major pandemic-era stages. Together, the selected intervals offer comparable duration and corpus size, providing a robust and contextually grounded basis for cross-phase emotional comparison.

### Ethical Considerations

Institutional ethics approval was not sought for this study because it involved only the secondary analysis of publicly available Reddit posts and did not involve recruitment, interaction, or intervention with human participants. The study was conducted in accordance with the ethical principles for internet-mediated research proposed by the Association of Internet Researchers [20], the International Code on Market, Opinion and Social Research and Data Analytics jointly issued by the International Chamber of Commerce and ESOMAR [21], and the recommendations of Eysenbach and Till for research involving publicly accessible internet communities [22].

This research relied exclusively on data available in the public domain of the Reddit platform. All data were sourced from the publicly accessible subreddit r/hemorrhoids, and the dataset was obtained from Pushshift, a public third-party data archive, ensuring that the research procedure was entirely noninvasive [23]. We programmatically deidentified all collected content and neither gathered nor retained any personally identifiable information, including usernames, user profile information, or IP addresses. Although usernames were present in the original Pushshift archive, they were removed during preprocessing so that no user-identifiable metadata was included in the analytical dataset.

To further protect user privacy, all analyses focused exclusively on aggregate discourse patterns and population-level trends rather than individual users or posts. Individual informed consent was not obtained because this study involved only the secondary analysis of deidentified, publicly available data without any recruitment or interaction with users [23]. The final dataset was securely stored on password-protected local devices and was accessible only to authorized members of the research team.

## Results

### Corpus Construction and Analytical Phasing

**Figure 1.**
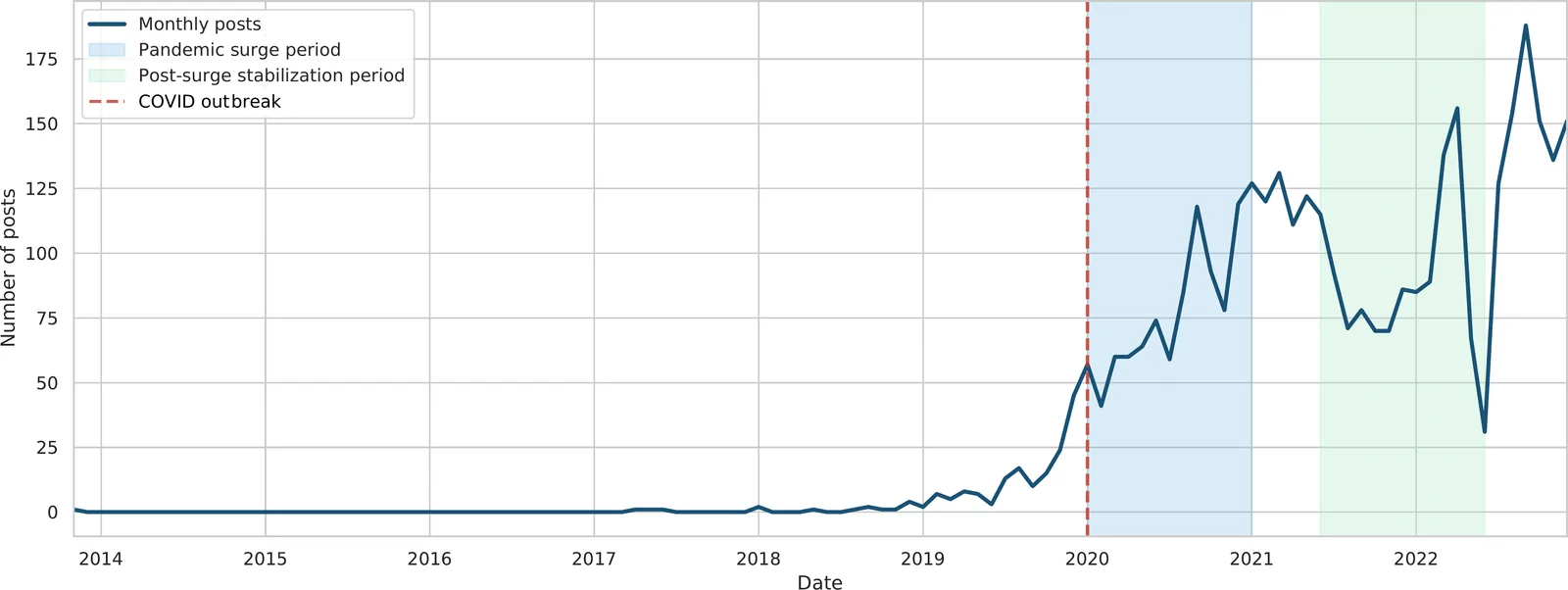
Time series analysis of monthly posting volume on r/hemorrhoids (2013‐2022).

Following data cleaning, the final corpus consisted of 3746 posts. Time series analysis of the monthly posting volume from 2013 to 2022 revealed 2 distinct developmental stages (Figure 1). The community began in an emergent phase (2013‐2018), characterized by minimal and stable activity, in which monthly post volumes rarely exceeded 1, establishing the baseline for future engagement. A decisive transition occurred in 2019, marking the onset of the mature phase (2019‐2022). This stage saw a sharp and sustained escalation in post frequency, accelerating dramatically in 2020. The subsequent period (2020‐2022) was defined by a consistently high, yet volatile, discourse environment. Analysis of this mature phase showed a rhythmic, recurring pattern of activity, with peak volumes concentrated in the first and fourth quarters of the year, typically followed by significant declines during the mid-year period (Q2 and Q3). This seasonal pattern recurred across consecutive years, suggesting a persistent intraannual rhythm in community activity.

### Overall Community Emotional Landscape

Multiclass emotion analysis of the entire corpus (N=3746) revealed a predominance of neutral emotion (n=1247, 33.29%), followed by fear (n=955, 25.49%), disgust (n=762, 20.34%), and sadness (n=450, 12.01%; Figure S2 and Table S1 in Multimedia Appendix 1). Joy accounted for a minimal fraction (n=26, 0.69%), indicating that expressions of positive affect were exceedingly rare within the discourse.

Further quantification revealed differences in community engagement across emotional categories (Figure S3 and Table S3 in Multimedia Appendix 1). Posts expressing joy achieved the highest mean score (5.69, SD 3.99), while those expressing fear and sadness elicited the highest mean comment counts (7.06, SD 7.60 and 7.03, SD 6.36, respectively), suggesting emotion-specific engagement dynamics.

### Topic Modeling Analysis: Delineating Core User Concerns

#### Overview

**Figure 2.**
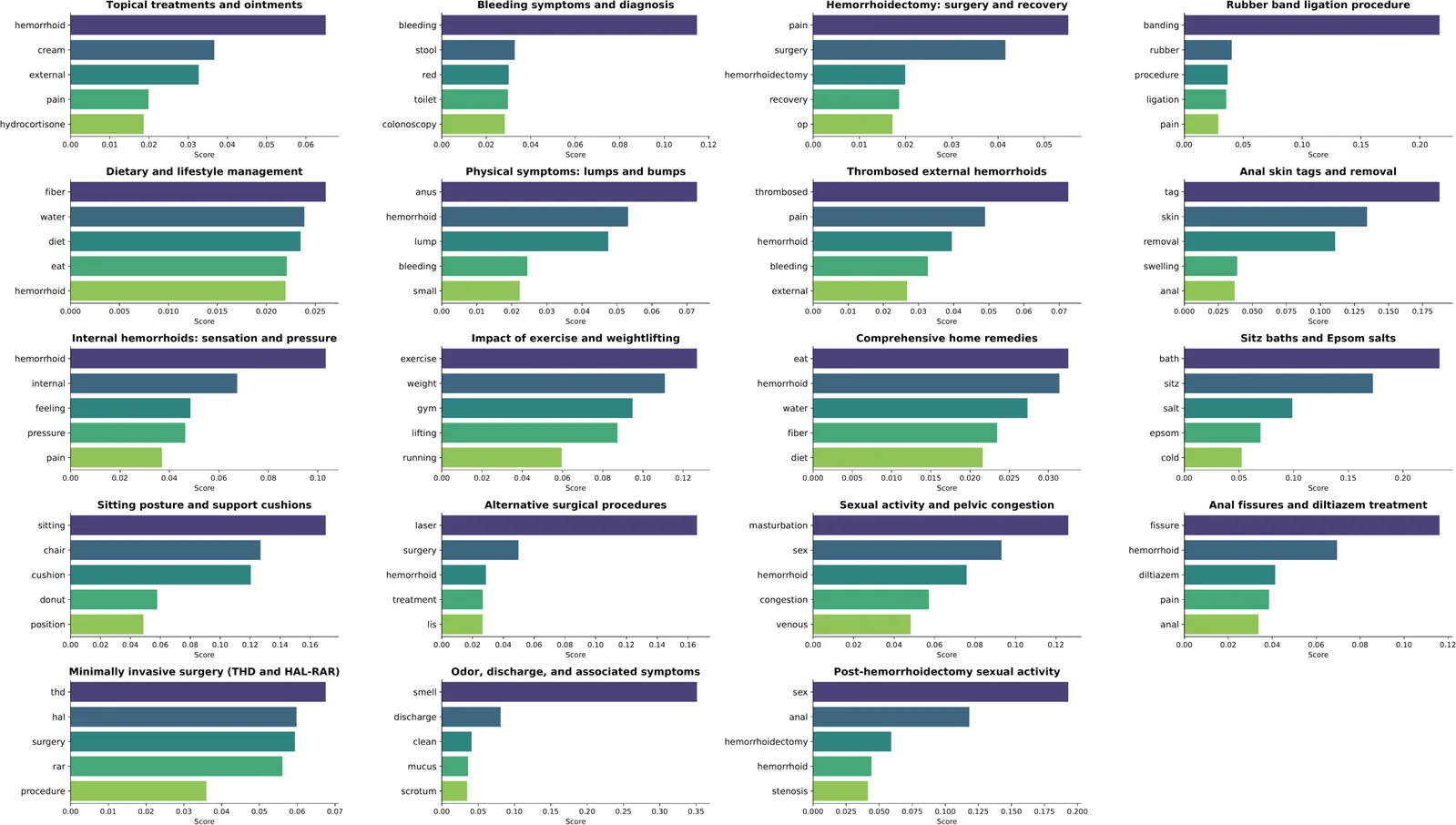
Top representative words and scores for identified topics in the mature-phase corpus.

Topic modeling of the mature-phase corpus (2019‐2022) identified 19 semantically coherent topics and 1370 outlier documents (topic −1). Quantitative evaluation of topic quality yielded a topic coherence (C_v) score of 0.489 and a topic diversity score of 0.695, providing additional support for the interpretability and distinctiveness of the identified topics. The proportion of outliers was consistent with typical outcomes of density-based clustering methods, which do not force low-density documents into semantic clusters. These topics were manually labeled through a consensus-based review, supported by large language model assistance (Table 1 and Figure 2). Analysis of topic frequency over time within this phase (Figure S6 in Multimedia Appendix 1) further revealed a dynamic and fluctuating evolution of user concerns, with themes related to “topical treatments and ointments,” “bleeding symptoms and diagnosis,” and “hemorrhoidectomy: surgery and recovery” consistently emerging as high-frequency topics, underscoring the community’s preoccupation with both conservative and invasive management strategies. For interpretive clarity, the topics were organized into 4 overarching thematic domains.

#### Domain I: Symptom Recognition and Diagnosis

This domain encompassed 5 topics dedicated to users’ identification and description of physiological symptoms. Key themes included “bleeding symptoms and diagnosis,” which focused on the characteristics of bleeding events and associated diagnostic procedures; “thrombosed external hemorrhoids,” centering on the acute pain and morphological features of this specific condition; “physical symptoms: lumps and bumps,” involving descriptions of perianal abnormalities; “internal hemorrhoids: sensation and pressure,” primarily concerning atypical discomforts such as tenesmus or prolapse sensation; and “odor, discharge, and associated symptoms,” addressing concerns related to hygiene and bodily secretions.

#### Domain II: Conservative Treatment and Self-Management

Represented by 4 topics, this domain reflected the noninvasive, home-based therapeutic modalities adopted by users. The most prevalent theme was “topical treatments and ointments,” which captured extensive user exchange regarding various over-the-counter products. Other themes included “dietary and lifestyle management,” focused on increasing dietary fiber intake; “comprehensive home remedies,” integrating multiple nonprescription strategies; and “sitz baths and Epsom salts,” emphasizing this core physical intervention.

#### Domain III: Medical and Surgical Interventions

Comprising 6 topics, this domain centered on clinical and operative management strategies. The most widely discussed theme was “hemorrhoidectomy: surgery and recovery,” with narratives spanning the entire process from preoperative preparation to postsurgical convalescence. Following this was the “rubber band ligation procedure,” covering procedural details and post-treatment experiences. Additional topics included the management of sequelae, such as “anal skin tags and removal”; discussions on concurrent pathologies, exemplified by “anal fissures and diltiazem treatment”; and explorations of both “alternative surgical procedures” and “minimally invasive surgery (transanal hemorrhoidal dearterialization and hemorrhoidal artery ligation and recto-anal repair).”

#### Domain IV: Quality of Life, Risk Factors, and Psychosocial Impact

The 4 topics in this final domain revealed the tangible impacts of the condition on users’ daily lives and well-being. Content included the discussion of specific behaviors as risk factors, such as the “impact of exercise and weightlifting” and “sitting posture and support cushions.” Furthermore, this domain addressed the effects on intimate life, with themes designated as “sexual activity and pelvic congestion” and “post-hemorrhoidectomy sexual activity.”

Across the 4 thematic domains, the 2360 nonoutlier posts assigned to the 19 identified topics were unevenly distributed. Domain I (symptom recognition and diagnosis) accounted for the largest proportion of posts (n=763, 32.33%), followed closely by Domain III (medical and surgical interventions; n=746, 31.61%) and Domain II (conservative treatment and self-management; n=711, 30.13%). Domain IV (quality of life, risk factors, and psychosocial impact) represented a substantially smaller share of topic-assigned posts (n=140, 5.93%).

### Structural and Semantic Topic Relationships

To ensure the interpretive robustness of the topic model and uncover the latent organization of user discourse, we conducted a multilayered structural and semantic validation.

### Document Cluster Distribution

**Figure 3.**
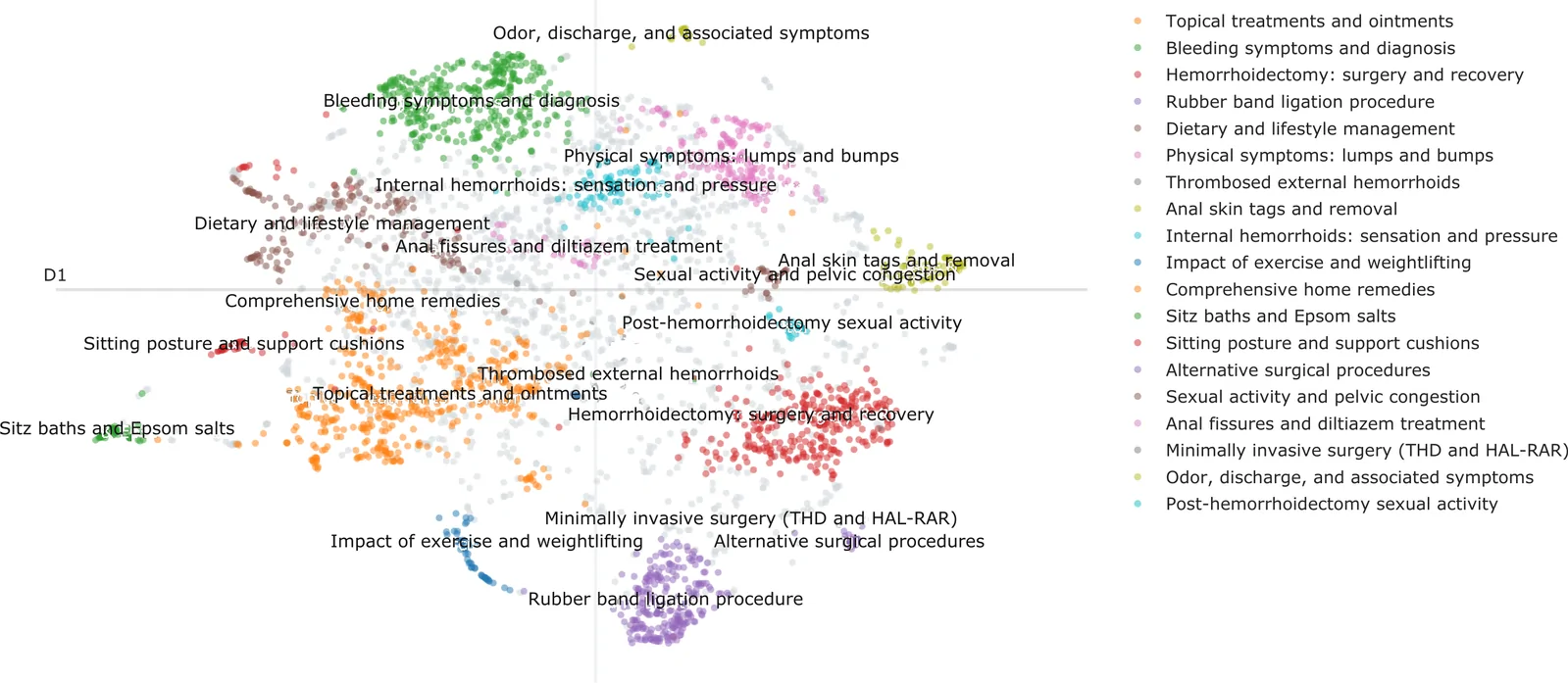
Uniform Manifold Approximation and Projection of document clusters. HAL-RAR: hemorrhoidal artery ligation and recto-anal repair; THD: transanal hemorrhoidal dearterialization.

The Uniform Manifold Approximation and Projection embedding (Figure 3) revealed well-defined, compact clusters corresponding to individual topics, highlighting the model’s strong intratopic semantic cohesion and clear intertopic separability. In contrast, documents classified as outliers (topic −1) were diffusely scattered across the embedding space, consistent with their interpretation as corpus-level noise.

### Intertopic Spatial Relationships

The intertopic distance map derived from multidimensional scaling (Figure S5 in Multimedia Appendix 1) revealed a distinctive “one-core, two-satellite” spatial configuration. Most themes coalesced into a dense central cluster in the fourth quadrant, encompassing discussions of core symptoms (eg, bleeding symptoms and diagnosis [topic 1]; thrombosed external hemorrhoids [topic 6]), self-management strategies (eg, topical treatments and ointments [topic 0]; dietary and lifestyle management [topic 4]), and mainstream medical interventions (eg, hemorrhoidectomy: surgery and recovery [topic 2]). A smaller peripheral cluster in the second quadrant was dominated by subjective symptom experiences (eg, physical symptoms: lumps and bumps [topic 5]; internal hemorrhoids: sensation and pressure [topic 8]; sexual activity and pelvic congestion [topic 14]), while an isolated cluster in the third quadrant captured technologically advanced or alternative procedures (eg, alternative surgical procedures [topic 13]; minimally invasive surgery [transanal hemorrhoidal dearterialization and hemorrhoidal artery ligation and recto-anal repair; topic 16]).

This structural differentiation suggests that users’ discussions naturally were organized around symptomatology, management practices, and surgical complexity.

### Topic Hierarchical Structure

**Figure 4.**
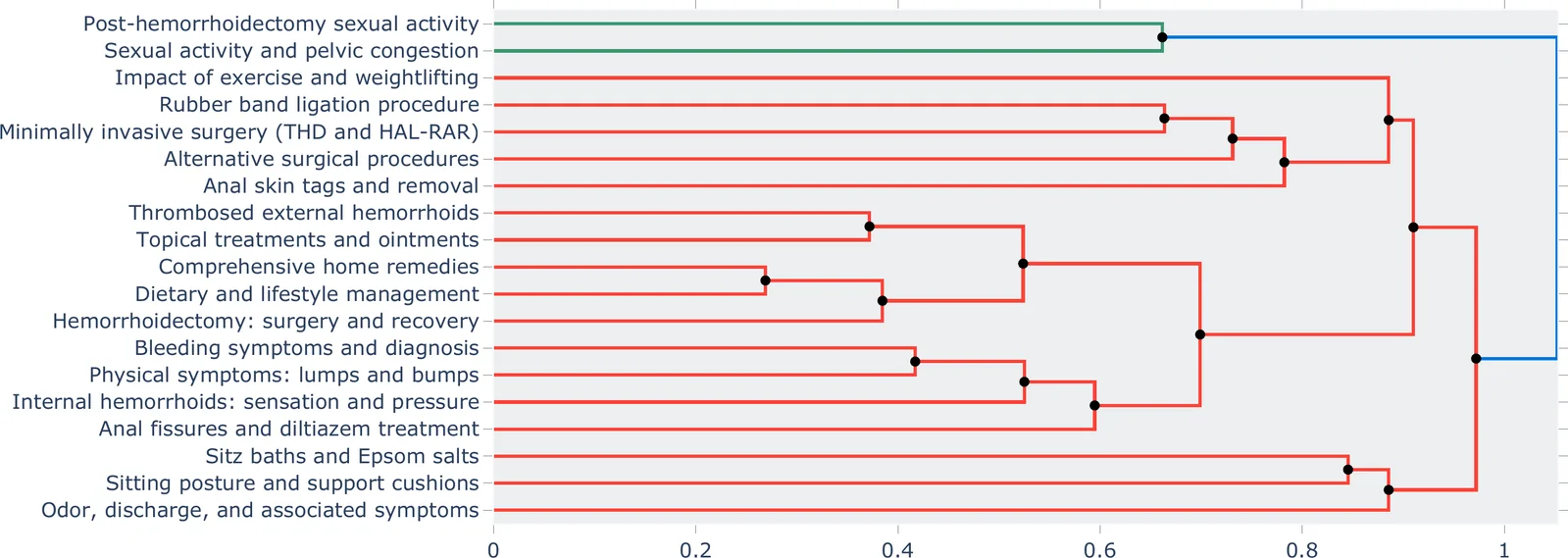
Dendrogram of thematic clusters showing binary hierarchical structure. HAL-RAR: hemorrhoidal artery ligation and recto-anal repair; THD: transanal hemorrhoidal dearterialization.

The hierarchical clustering analysis (Figure 4) further validated the robustness of the thematic taxonomy, revealing a binary top-level bifurcation. One specialized branch was dedicated to discussions of sexual activity (topics 14 and 18), whereas the remaining 17 topics formed a supercluster encompassing all other domains. Within this supercluster, a secondary split clearly separated conservative self-management themes (eg, topical treatments [topic 0]; diet [topic 4]; and sitz baths [topic 11]) from surgical interventions (eg, hemorrhoidectomy [topic 2]; ligation [topic 3]; and skin tag removal [topic 7]).

### Topic Similarity Matrix

**Figure 5.**
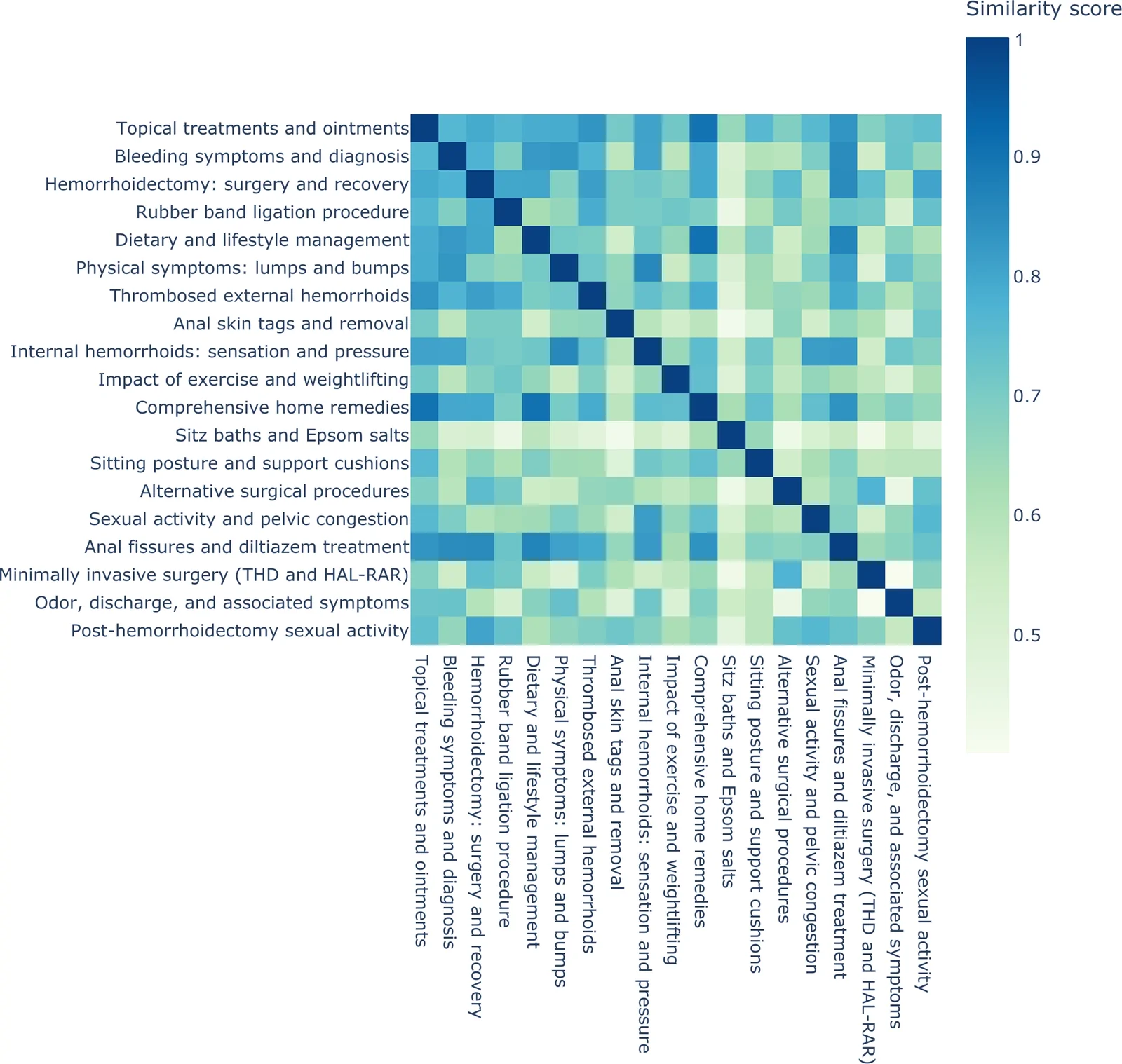
Heatmap of intertopic semantic similarity (cosine similarity). HAL-RAR: hemorrhoidal artery ligation and recto-anal repair; THD: transanal hemorrhoidal dearterialization.

To assess the semantic relationships among topics, we calculated pairwise cosine similarities between topic embeddings and visualized the results in a heatmap (Figure 5). Darker cells represent greater similarity, which was primarily observed among conceptually related topics such as topical treatments and ointments (topic 0), comprehensive home remedies (topic 10), and dietary and lifestyle management (topic 4). A notable correlation was also detected between anal fissures and diltiazem treatment (topic 15) and dietary and lifestyle management (topic 4). In contrast, thematically distant topics, including minimally invasive surgery (topic 16) and odor, discharge, and related symptoms (topic 17), exhibited low similarity values. Overall, this distinct pattern of high within-cluster and low between-cluster similarity provided quantitative support for the semantic coherence and discriminant validity of the model’s topic structure.

### Analysis of Emotional Dynamics

#### Community Emotional Distribution

Multiclass emotion classification of the corpus (N=3746) revealed a heterogeneous emotional landscape. Neutral sentiment predominated (n=1247, 33.29%), followed by substantial proportions of fear (n=955, 25.49%) and disgust (n=762, 20.34%; Figure S2 and Table S1 in Multimedia Appendix 1). This distribution suggests that the community simultaneously served as a space for factual information exchange (neutral expressions) and as an outlet for health-related anxiety and physical aversion (fear and disgust). Sadness constituted a smaller yet notable share (n=450, 12.01%), whereas joy accounted for only 26 posts (0.69%). The scarcity of positive affective expressions highlighted the predominance of distress-oriented communication within the forum.

#### Topic-Emotion Association

**Figure 6.**
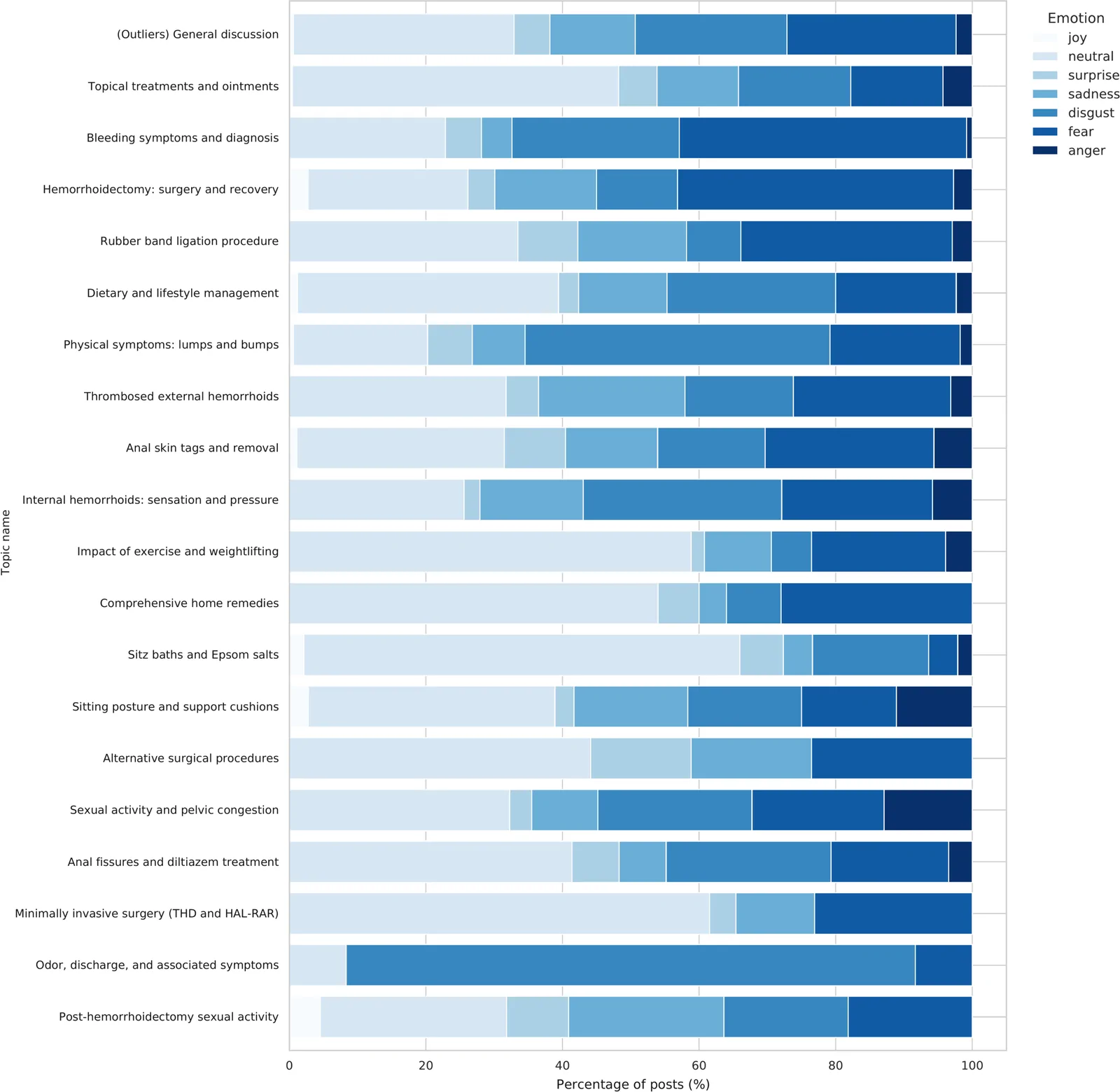
Emotional distribution across topics (topic-emotion cross-analysis). HAL-RAR: hemorrhoidal artery ligation and recto-anal repair; THD: transanal hemorrhoidal dearterialization.

Topic-emotion association analysis (Figure 6 and Table S2 in Multimedia Appendix 1) revealed distinct emotional profiles across topics. Fear was most prominent in discussions involving medical interventions and acute symptoms, reaching its highest levels in hemorrhoidectomy (fear: 133/329, 40.4%) and bleeding symptoms and diagnosis (fear: 151/359, 42.1%). Disgust was strongly associated with physical manifestations of the condition, particularly in physical symptoms: lumps and bumps (75/168, 44.6%). Sadness was most frequent in post-hemorrhoidectomy sexual activity (5/22, 22.73%). In contrast, self-management topics such as topical treatments and ointments (neutral: 212/444, 47.8%) and dietary and lifestyle management (neutral: 65/170, 38.2%) showed a higher proportion of neutral sentiment, suggesting a primarily informational rather than emotional purpose.

#### Emotional Impact on Community Engagement

Community engagement, measured by average post scores and comment counts (Figures S3 and S4 and Table S3 in Multimedia Appendix 1), differed substantially across emotional categories. Posts expressing joy received the highest mean post score of 5.69 (SD 3.99) and had a mean comment count of 6.54 (SD 7.10). Posts expressing fear and sadness elicited the highest mean comment counts (7.06, SD 7.60 and 7.03, SD 6.36, respectively), indicating that negatively valenced emotions, particularly anxiety and distress, tend to generate greater community interaction. In contrast, posts expressing disgust or neutral sentiment achieved lower engagement levels overall, reflecting a more informational rather than interactive communication pattern.

#### Diurnal Rhythm of Emotional Expression

**Figure 7.**
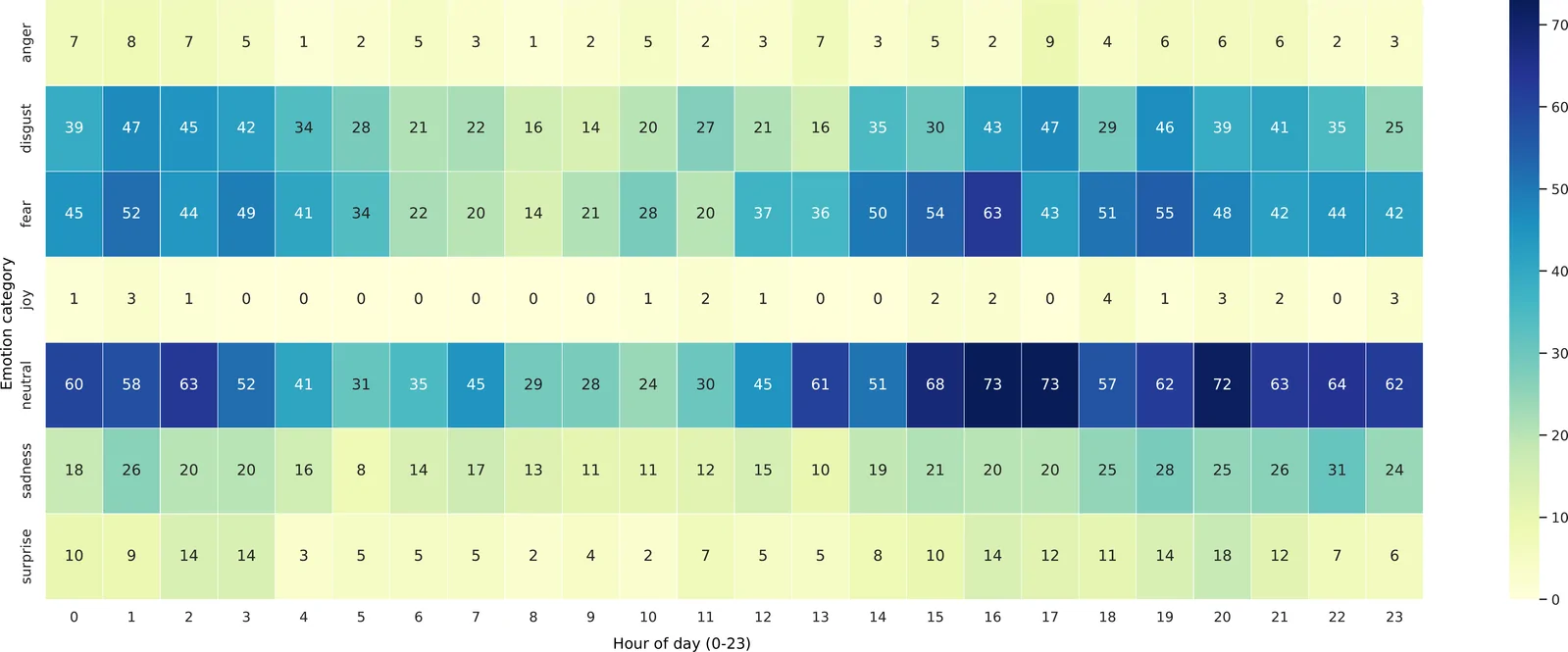
Diurnal rhythm of emotional expression in hemorrhoid discussions.

Temporal analysis revealed a clear diurnal pattern in emotional expression (Figure 7). In reference to North American and European time zones, neutral sentiment peaked during daytime hours (2 PM to 8 PM UTC). Negative emotions, including fear, sadness, and disgust, were more frequent at night (9 PM to 5 AM UTC). This shift suggests that users tend to seek information during the day and emotional support during late hours.

#### Emotion Distribution Across Pandemic Phases

Comparative analyses between the pandemic surge period (January 2020 to January 2021) and the post-surge stabilization period (June 2021 to June 2022) indicated that the overall emotional profile of community discussions remained remarkably consistent across the 2 intervals. In both periods, neutral expressions accounted for approximately 33% to 35% of all posts, followed by fear (23%-27%) and disgust (18%-22%; Figure S7 and Table S4 in Multimedia Appendix 1).

Although the general pattern was stable, several modest shifts were observed. The prevalence of fear increased slightly by 3.52 percentage points in the later period, while both disgust and neutral expressions declined by 2.82 and 1.87 percentage points, respectively. Other emotional categories, including sadness, surprise, anger, and joy, exhibited only negligible changes, all well below 1 percentage point. These results suggest a relatively stable emotional landscape across the 2 pandemic-related periods, despite substantial differences in posting volume and external social context.

## Discussion

### Principal Findings

This study presents a longitudinal computational linguistic analysis of the Reddit hemorrhoids community, combining analyses across the full study period (2013‐2022) with topic modeling focused on the community’s mature phase (2019‐2022). Our primary aim was to systematically deconstruct the discourse structure, functional evolution, and underlying emotional dynamics of this stigmatized illness in the OHC. By applying advanced transformer-based models for topic identification and sentiment quantification, our empirical findings contribute to the limited literature regarding public discourse analysis of anorectal diseases and provide novel, data-driven insights into the crucial supportive role of user-generated content in chronic disease management and the health journey in the digital age.

At the domain level, discussions spanned symptom recognition, conservative self-management, and medical or surgical intervention domains in broadly comparable proportions, while quality of life and psychosocial issues appeared less frequently as stand-alone topics. Our analysis clearly defined the r/hemorrhoids community as a support-oriented ecosystem underpinned by a dual core functionality: the coexistence of instrumental information exchange and socioemotional support [24]. On the cognitive and informational level, the 19 core topics identified by our topic model delineated a systemic and traceable trajectory of clinical decision-making, from symptom discovery to clinical intervention. The identified topics broadly reflected the patient illness journey, spanning symptom recognition, self-management strategies, and surgical interventions. This thematic organization is broadly consistent with previous computational analyses of Reddit-based health communities, which similarly found that patient discussions tend to center on symptom appraisal, self-management, treatment decision-making, and peer experience sharing [25,26]. However, compared with these broader health communities, the hemorrhoid community exhibited a particularly coherent progression from symptom recognition to treatment decisions, reflecting the structured illness trajectory experienced by patients with a stigmatized anorectal condition. This structured organization suggests that the community may function as a shared repository of illness trajectory knowledge, with longitudinal user exchanges documenting common patterns of symptom progression and illness management. Crucially, the hierarchical clustering and semantic similarity matrix further validated this intrinsic logical structure, where the clear bifurcation between “conservative treatment” and “surgical intervention” discussions accurately reflected the 2-phased and often mutually exclusive decision sets patients face in disease management. This finding deepens our understanding of OHC functional classification, revealing how structured user-generated content can construct a knowledge layer that simulates or even complements primary medical guidance. However, the accuracy and safety of community-generated advice are beyond the scope of this study and warrant future investigation. On the emotional and psychosocial expression level, the community exhibited an overall negative emotional tone dominated by fear, disgust, and sadness, which is highly consistent with the societal stigma and psychological burden associated with hemorrhoids [5,27]. The predominance of fear and other negative emotions is also consistent with previous Reddit studies showing that users of health-related support communities primarily use these platforms to express uncertainty, distress, and illness-related concerns [26,28]. Compared with mental health communities, however, the emotional expressions observed in our study were more closely linked to concrete symptoms, treatment decisions, and bodily stigma rather than generalized psychological distress. A finer-grained topic-emotion analysis further revealed that emotional expression was highly context dependent: fear was particularly prominent in topics related to acute symptoms and invasive interventions, highlighting users’ profound anxieties regarding the high-stakes indicator of “bleeding” and the high-morbidity procedure of “hemorrhoidectomy” [29,30]. This confirms the community’s core value as a risk perception buffer and health anxiety outlet during these critical decision junctures. Simultaneously, disgust peaked in themes describing physical symptoms (eg, “lumps and bumps”), powerfully underscoring the bodily stigma imposed by the disease on physical appearance and personal hygiene; the anonymous online environment may function as a safe harbor for users to openly articulate core physical distress that is often unspeakable in real-life social settings [31]. The anonymous nature of the platform may also facilitate emotional disclosure and the normalization of stigmatized experiences, consistent with theories of stigma management and resilience [32]. In contrast to negative emotions, neutral sentiment dominated self-management topics (eg, “topical treatments”), indicating that for these low-risk, actionable issues, the community’s discourse function is primarily concentrated on de-emotionalized objective knowledge sharing and efficacy assessment [33].

Our analysis of the association between emotional content and community engagement (measured by post score and comment count) provides insights into engagement patterns within OHCs. The results indicated that despite fear being the second-largest emotional category, posts expressing joy garnered the highest mean scores, while those expressing fear and sadness attracted the most comments—suggesting distinct patterns of score-based endorsement and comment-based interaction. This finding is broadly consistent with previous studies demonstrating that emotionally salient posts tend to elicit greater user interaction within OHCs [26,34]. However, unlike some prior studies that interpreted increased commenting primarily as evidence of peer support, we adopt a more cautious interpretation because comment activity may also reflect symptom severity, uncertainty, or requests for advice. Specifically, 2 complementary engagement patterns emerged within the OHC. First, a pattern of positive reinforcement surrounding recovery-related posts: users expressing positive treatment outcomes or recovery experiences garnered the highest average scores, suggesting greater positive endorsement of recovery-related experiences within the community. This suggests a collective desire for success stories, whereby members seek emotional efficacy from recovery narratives to offset the disease’s negative impact [35,36]. Second, an engagement pattern associated with posts expressing sadness or distress: sad posts elicited among the highest mean comment counts despite having comparatively modest mean post scores. This pattern suggests that posts expressing pain or helplessness tend to generate greater community interaction. While such engagement may reflect supportive responses and emotional resonance, comment quantity alone cannot determine the nature or quality of these interactions and may also reflect heightened symptom severity, uncertainty, or requests for advice. Nevertheless, the finding highlights the potential role of anonymous forums as spaces where individuals with stigmatized illnesses seek interaction, information, and potentially supportive exchanges with peers [37,38]. Complementing these dynamics, the BASE (belonging, affirmation, safety, and efficacy) model further suggests that humor, often observed in the qualitative discourse, functions as an affective moderator in the community’s emotional ecosystem. Users often use self-deprecating or absurdist humor to simultaneously express vulnerability and reclaim agency. Such humor-driven reframing enhances peer bonding, diffuses anxiety, and sustains collective resilience under the chronic stigma pressure surrounding anorectal disorders [32]. Furthermore, the observed diurnal rhythm, with negative emotions concentrated during the late evening and overnight hours, suggests a potential late-night window for emotional disclosure. This temporal gradient suggests that users turn to the OHC for implicit support primarily when they are disengaged from daily social and professional pressures and are confronting their illness in solitude [37,39]. This pattern may inform future research on timing-sensitive digital support or remote interventions.

Despite the broad psychosocial disruptions associated with COVID-19, the emotional profile of the community remained relatively stable across pandemic phases. This finding contrasts with several Reddit-based studies of broader mental health and disease-specific communities, in which the pandemic substantially altered discussion topics and emotional expression [40,41]. One possible explanation is that hemorrhoid-related discussions are driven primarily by persistent disease-specific concerns rather than acute external events, resulting in comparatively stable emotional patterns across pandemic phases. Together, these findings suggest that discussions within condition-specific OHCs may be shaped more strongly by enduring illness-related stressors than by transient societal events, although community engagement remained active throughout the pandemic [42,43].

This study offers theoretical and practical insights for clinical communication, digital health design, and computational social science.

Clinically, the cooccurrence of anxiety-related themes (bleeding and surgery) and sadness indicates strong unmet needs for reassurance and empathy. Clinicians should address patients’ implicit fears and integrate emotional validation into consultations to strengthen shared decision-making. The community’s active exchange of self-management information highlights OHCs as an extended domain of patient education; clinicians can engage with these spaces to guide patients toward evidence-based practices [44].

For digital health, the findings inform emotionally responsive and context-aware design. Frequent discussions of sexuality and lifestyle revealed psychosocial needs often omitted in clinical narratives. Digital platforms can leverage topic-emotion associations to deliver tailored, anxiety-reducing messages and use shame-resilient, humor-informed designs to promote belonging and safety, aligning with the BASE framework [32]. However, these findings should not be interpreted as justification for algorithmically amplifying distress-related content to increase engagement. Future research should examine how online health platforms can support emotionally vulnerable users while minimizing risks of manipulation, emotional exploitation, or unintended reinforcement of negative affect. Methodologically, the integration of BERTopic and fine-grained sentiment analysis demonstrates the strength of transformer-based models in capturing nuanced, affective discourse. This framework establishes a benchmark for future OHC research, advancing from surface-level text mining toward deep semantic analysis.

This study has several limitations. It relied on a single platform, Reddit, which may limit the generalizability of the findings to other online contexts. In addition, although no obvious evidence of widespread automated activity was observed, the presence of undetected bot-generated content cannot be completely excluded and may have influenced a small portion of the analyzed posts. Topic modeling was restricted to the community’s mature phase (2019‐2022) because of limited posting activity in earlier years. Consequently, the thematic findings may not fully capture discourse patterns during the community’s formative period. Moreover, BERTopic classified a substantial proportion of posts as outliers. Although this is a common feature of density-based clustering methods, excluding these posts may have reduced the representation of niche, highly individualized, or less coherent discussions within the final topic structure. Furthermore, broader cultural and societal changes between 2013 and 2022, including shifts in online communication practices, health awareness, and disease-related stigma, may have affected patterns of community engagement and were not explicitly controlled for in this study. The sentiment analysis may also have introduced minor bias due to the truncation of long posts. In addition, the emotion classification model used in this study was a general-purpose pretrained model rather than one specifically trained on health-related Reddit discourse. As a result, nuanced expressions common in OHCs, such as sarcasm, embarrassment, humor, or stigmatized language, may not always be accurately captured, potentially introducing some degree of misclassification. Topic interpretation inevitably involves some degree of subjectivity despite human and model-based validation. Future research should extend this framework to cross-platform comparisons to examine how different digital environments shape stigmatized illness discourse and social support. Longitudinal modeling of emotional dynamics could further illuminate how sentiment evolves across disease trajectories and clinical events. Additionally, qualitative analysis of outlier posts may uncover marginalized yet important patient experiences, including the role of humor in coping and identity reconstruction. Future work should also examine how OHCs can maintain supportive responses to emotional disclosures while mitigating potential misuse, including attention-seeking or manipulative behaviors. Overall, this study provides a computational lens for understanding the emotional and structural foundations of support in stigmatized health communities, offering evidence for more empathetic and inclusive digital health design.

### Conclusions

This study provides one of the first longitudinal computational analyses of a stigmatized illness community using Reddit data, revealing how collective discourse on hemorrhoids evolved into a structured knowledge base that integrates emotional support with practical self-management. Fear, sadness, and disgust dominated the emotional landscape, and posts expressing fear or sadness were associated with higher comment activity. By combining transformer-based topic and sentiment modeling, this work demonstrates how advanced NLP can help characterize thematic and emotional patterns within online patient communities. These findings underscore the potential of OHCs as critical infrastructures for empathy-centered communication, participatory education, and inclusive digital health design.

## Supplementary material

10.2196/90427 Multimedia Appendix 1 Additional tables and figures on data preprocessing, topic modeling, emotional distributions, topic-emotion associations, community engagement, and temporal analyses.

## Abbreviations

BASE: belonging, affirmation, safety, and efficacy
c-TF-IDF: class-based term frequency–inverse document frequency
NLP: natural language processing
OHC: online health community
