# Supplementary material for: Evolution of Discourse in the Reddit Hemorrhoid Community: Topic and Sentiment Analysis
Source: J Med Internet Res. 2026 Sep 21;28:e90427. doi: 10.2196/90427 (PMC13592657; doi:10.2196/90427)
Supplement: Supplementary file 1 [file jmir-v28-e90427-s001.pdf]

Supplementary material

Contents

Table S1. Percentage distribution of emotional categories in the full corpus

Table S2. Percentage distribution of emotional categories by topic

Table S3. Mean community engagement metrics by emotional category

Table S4. Emotion distributions and percentage-point differences between the pandemic surge and post-surge stabilization periods

Figure S1. Data preprocessing and screening flowchart

Figure S2. Emotional distribution of the r/hemorrhoids community corpus

Figure S3. Mean post score across emotional categories

Figure S4. Mean number of comments across emotional categories

Figure S5. Multidimensional scaling (MDS) map of intertopic spatial relationships

Figure S6. Temporal evolution of topic frequency during the mature phase of the r/hemorrhoids community

Figure S7. Comparative emotion distributions across the pandemic surge and post-surge stabilization periods

Table S1. Percentage distribution of emotional categories in the full corpus

Emotion	Percentage (%)
Neutral	33.29
Fear	25.49
Disgust	20.34
Sadness	12.01
Surprise	5.39
Anger	2.78
Joy	0.69

Table S2. Percentage distribution of emotional categories by topic

Topic name	Joy	Neutral	Surprise	Sadness	Disgust	Fear	Anger
(Outliers) General discussion	0.58	32.32	5.27	12.48	22.22	24.75	2.38
Topical treatments and ointments	0.45	47.75	5.63	11.94	16.44	13.51	4.28
Bleeding symptoms and diagnosis	0	22.84	5.29	4.46	24.51	42.06	0.84
Hemorrhoidectomy: surgery and recovery	2.74	23.4	3.95	14.89	11.85	40.43	2.74
Rubber band ligation procedure	0	33.47	8.79	15.9	7.95	30.96	2.93
Dietary and lifestyle management	1.18	38.24	2.94	12.94	24.71	17.65	2.35
Physical symptoms: lumps and bumps	0.6	19.64	6.55	7.74	44.64	19.05	1.79
Thrombosed external hemorrhoids	0	31.75	4.76	21.43	15.87	23.02	3.17
Anal skin tags and removal	1.12	30.34	8.99	13.48	15.73	24.72	5.62
Internal hemorrhoids: sensation and pressure	0	25.58	2.33	15.12	29.07	22.09	5.81
Impact of exercise and weightlifting	0	58.82	1.96	9.8	5.88	19.61	3.92
Comprehensive home remedies	0	54	6	4	8	28	0
Sitz baths and Epsom salts	2.13	63.83	6.38	4.26	17.02	4.26	2.13
Sitting posture and support cushions	2.78	36.11	2.78	16.67	16.67	13.89	11.11
Alternative surgical procedures	0	44.12	14.71	17.65	0	23.53	0
Sexual activity and pelvic congestion	0	32.26	3.23	9.68	22.58	19.35	12.9
Anal fissures and diltiazem treatment	0	41.38	6.9	6.9	24.14	17.24	3.45
Minimally invasive surgery (THD and HAL-RAR)	0	61.54	3.85	11.54	0	23.08	0
Odor, discharge, and associated symptoms	0	8.33	0	0	83.33	8.33	0
Post-hemorrhoidectomy sexual activity	4.55	27.27	9.09	22.73	18.18	18.18	0

Note. The "(Outliers) General discussion" category includes all non-topic-assigned posts (n=1386): 1370 mature-phase posts classified as topic -1 by BERTopic and 16 early-stage posts that were not included in topic modeling. Values are percentages.

Table S3. Mean community engagement metrics by emotional category

Emotion	Post score, mean (SD)	Number of comments, mean (SD)
Anger	3.29 (4.27)	6.02 (5.43)
Disgust	2.98 (3.44)	5.55 (5.38)
Fear	3.58 (3.90)	7.06 (7.60)
Joy	5.69 (3.99)	6.54 (7.10)
Neutral	3.11 (3.46)	5.92 (6.64)
Sadness	3.33 (3.00)	7.03 (6.36)
Surprise	3.26 (4.41)	5.89 (6.34)

Note. Values are mean (SD). SD: standard deviation.

Table S4. Emotion distributions and percentage-point differences between the pandemic surge and post-surge stabilization periods

Emotion	Pandemic surge period (Jan 2020–Jan 2021)	Post-surge stabilization period (Jun 2021–Jun 2022)	Difference
Neutral	35.17	33.3	1.87
Fear	23.93	27.45	-3.52
Disgust	21.71	18.88	2.82
Sadness	10.56	10.75	-0.19
Surprise	5.43	6.12	-0.69
Anger	2.91	3.06	-0.15
Joy	0.29	0.44	-0.15

Note. The analytic windows used end-exclusive upper date boundaries. Values are percentages; differences are percentage points (pandemic surge period minus post-surge stabilization period).

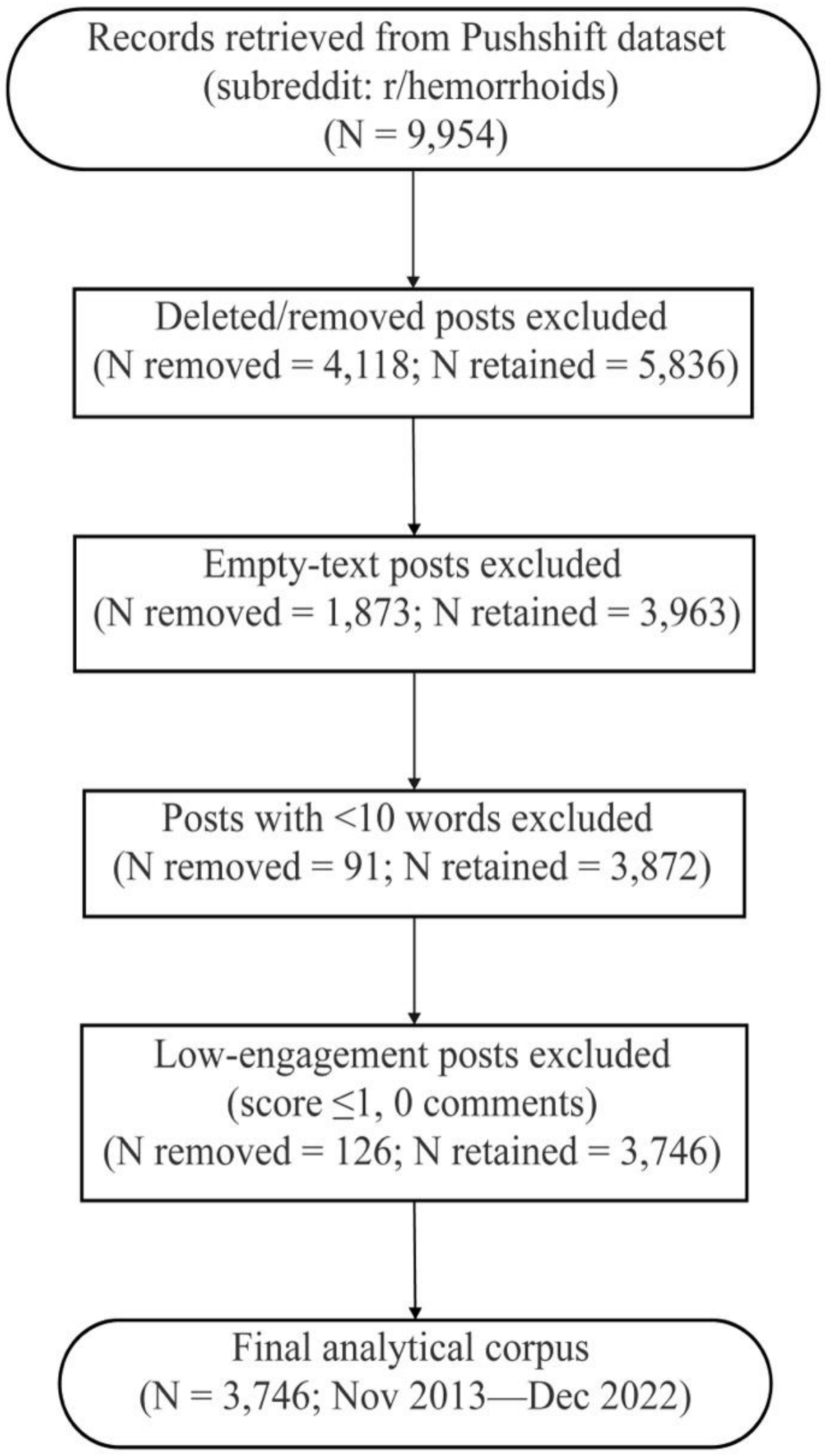

Figure S1. Data preprocessing and screening flowchart

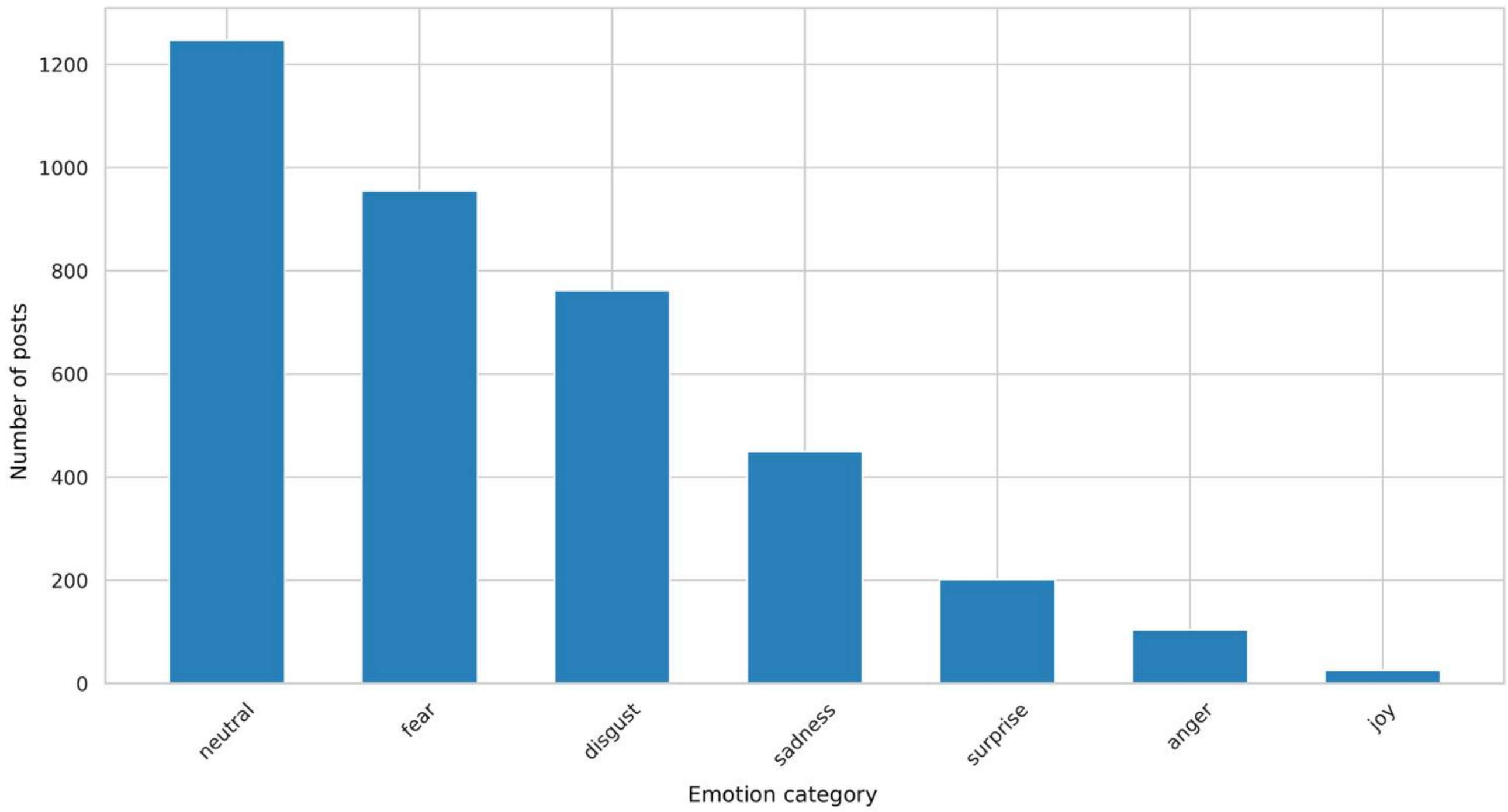

Figure S2. Emotional distribution of the r/hemorrhoids community corpus

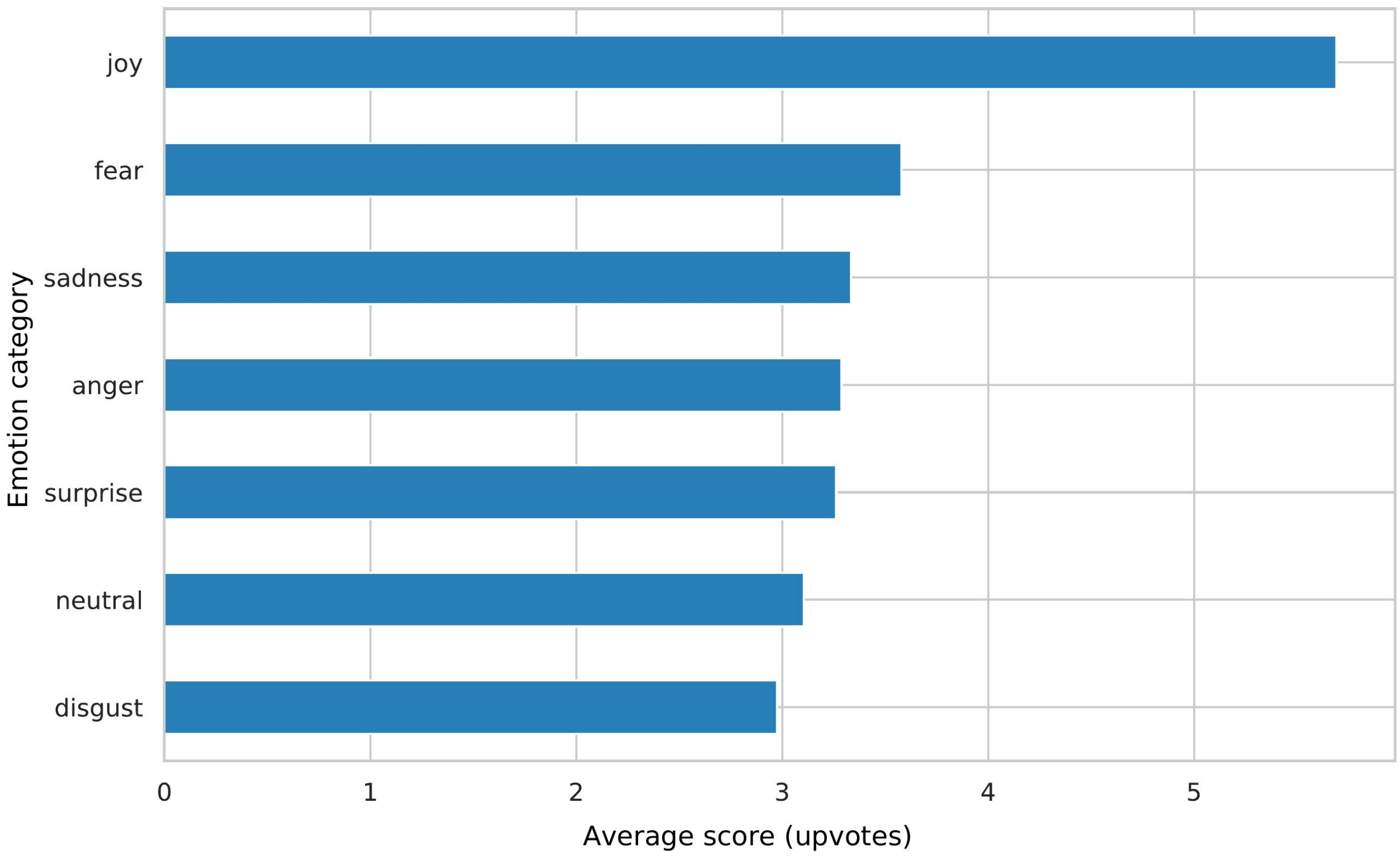

Figure S3. Mean post score across emotional categories

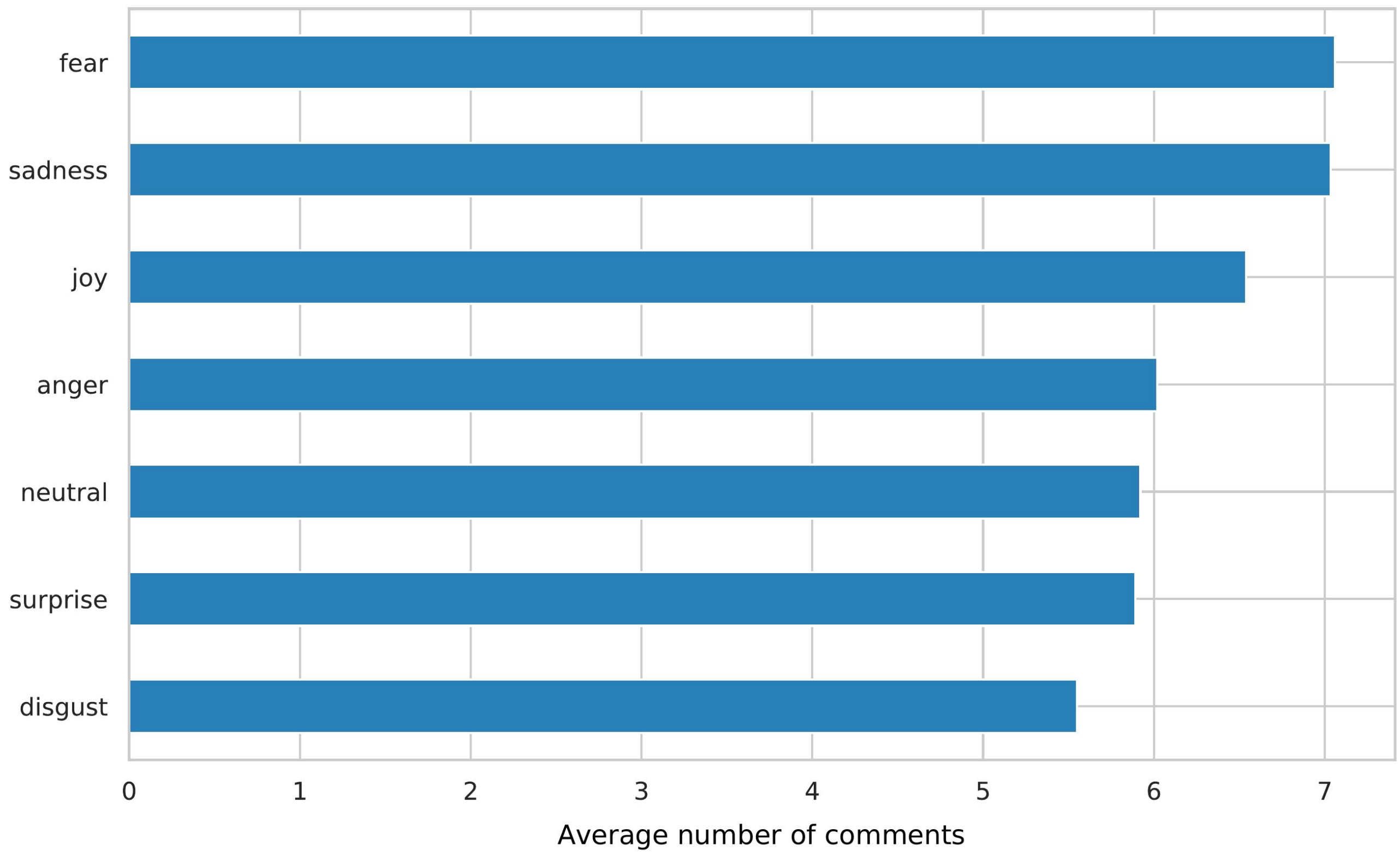

Figure S4. Mean number of comments across emotional categories

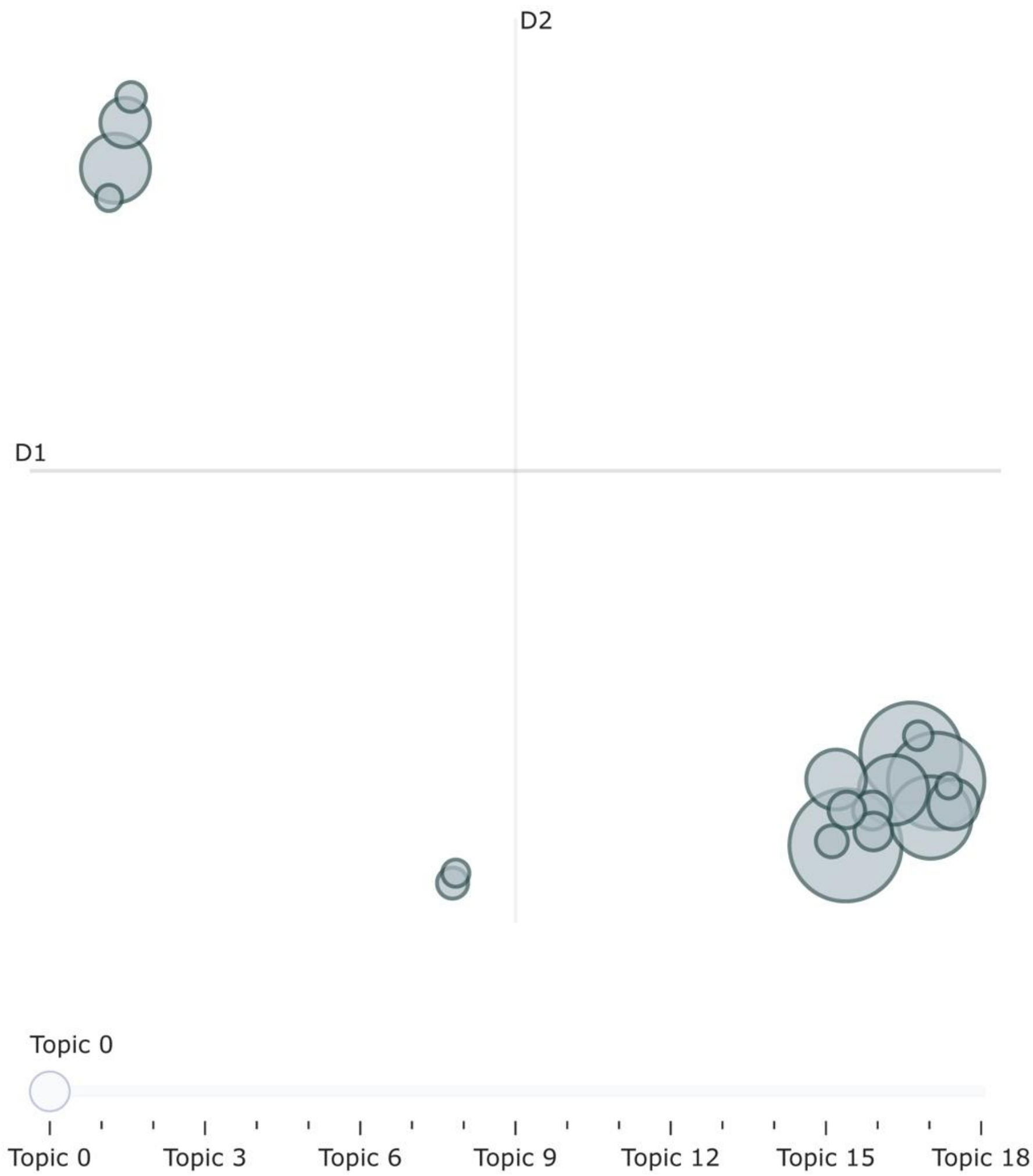

Figure S5. Multidimensional scaling (MDS) map of intertopic spatial relationships

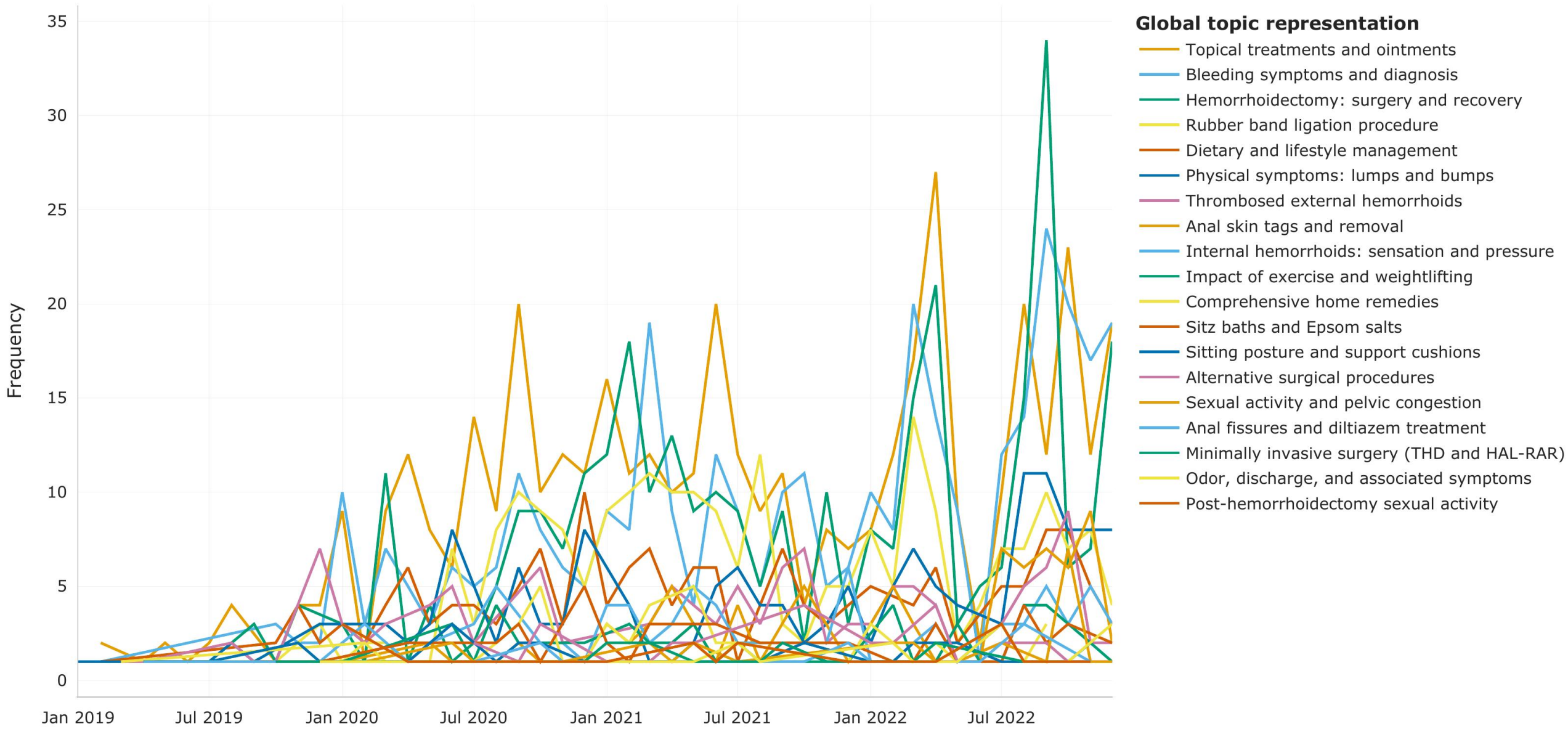

Figure S6. Temporal evolution of topic frequency during the mature phase of the r/hemorrhoids community

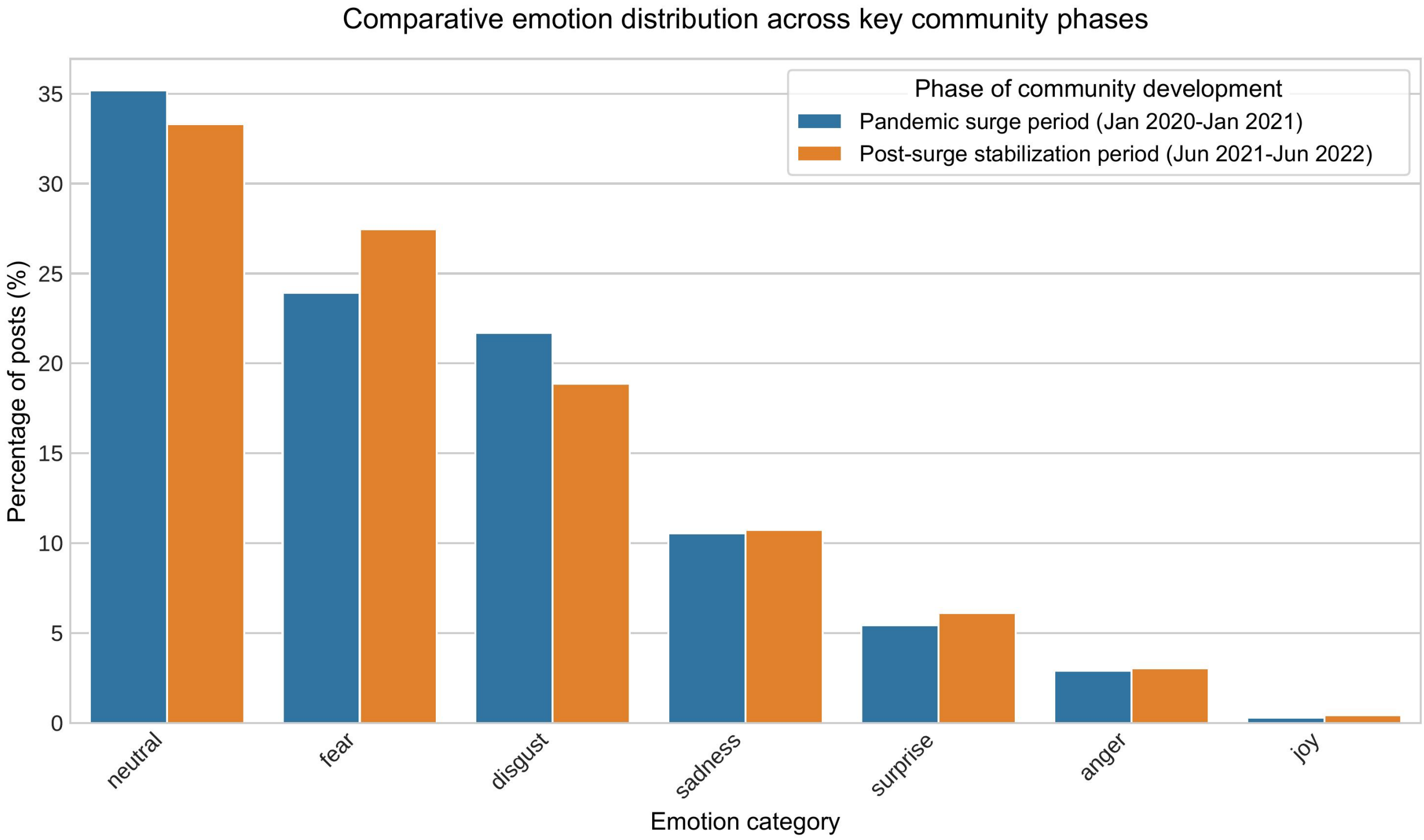

Figure S7. Comparative emotion distributions across the pandemic surge and post-surge stabilization periods
